# Ebola Virus Disease Preparedness in Subnational Health Systems: A Readiness Assessment of Jinja District, Uganda

**DOI:** 10.1155/jotm/5519966

**Published:** 2026-02-10

**Authors:** Joseph Oposhia, Joseph M. Kungu, Peter Dyogo Nantamu, Josephine Namayanja, Charles A. B. Okuyo, Michael Mulowoza, Kenneth Kabali, Katushabe Edson, Peter Olupot-Olupot

**Affiliations:** ^1^ Department of Community Health, Infectious Diseases Field Epidemiology and Biostatistics in Africa Fellowship Program, Faculty of Health Science, Busitema University, Mbale, Uganda, busitema.ac.ug; ^2^ Department of Health, Jinja District Local Government, Jinja, Uganda; ^3^ Department of Public Health, Busoga Public Health Emergency Operations Centre, Jinja Regional Referral Hospital, Ministry of Health, Jinja, Uganda, behdasht.gov.ir; ^4^ Department of Animal Resources and Biosecurity, College of Veterinary Medicine, Makerere University, Kampala, Uganda, mak.ac.ug; ^5^ School of Doctoral Studies, Unicaf University, Lusaka, Zambia; ^6^ Department of Animal Health, Animal Industry and Fisheries, Ministry of Agriculture, Entebbe, Uganda, agriculture.tn; ^7^ Emergency Preparedness and Response Cluster, World Health Organization Uganda Country Office, Kampala, Uganda

**Keywords:** community engagement, coordination, infection prevention and control (IPC), readiness, targeted interventions

## Abstract

**Background:**

Ebola virus disease (EVD) remains a significant public health threat in sub‐Saharan Africa. Jinja District in Uganda has experienced two EVD outbreaks in the recent past, first in November 2022 and again in February 2025, positioning it among the country’s EVD hotspots during Uganda’s eight recorded outbreaks. This study assessed the readiness of healthcare facilities by identifying existing gaps and strengths and providing evidence to inform targeted interventions to strengthen emergency preparedness and response.

**Methods:**

A cross‐sectional study was done using the WHO EVD readiness checklist. Data were collected through observations, interviews, and document reviews, and indicators were scored accordingly. Thematic analysis was used to summarize strengths and weaknesses and to categorize EVD readiness response based on indicator scores.

**Results:**

A total of 36 healthcare facilities were assessed in Jinja District, yielding an overall district EVD readiness score of 82%. Among the key indicators, coordination scored highest at 93%, while community engagement scored lowest at 77%. Based on facility‐level assessments, 20 facilities (55.6%) demonstrated high EVD readiness, 7 (19.4%) had medium readiness, and 9 (25%) showed low readiness. At the facility level, average scores across indicators were coordination (97.2%), surveillance (86.1%), case management and infection prevention and control (85.7%), community engagement (71.4%), logistics and supply chain (65.7%), and laboratory systems (60.0%).

**Conclusion:**

The assessment shows encouraging levels of Ebola readiness in a majority of Jinja District facilities, especially in coordination and surveillance. However, noticeable gaps remain in community engagement, IPC implementation, logistics, and laboratory systems, especially among low‐ and medium‐performing facilities. These findings highlight the need for targeted support, regular assessments, supply chain strengthening, and continuous capacity‐building to ensure all facilities can effectively respond to future EVD threats.

## 1. Introduction

Ebola virus disease (EVD) outbreaks continue to persist as a major public health emergency in sub‐Saharan Africa. EVD is a severe and often fatal viral hemorrhagic fever caused by a thread‐shaped virus of the family *Filoviridae* [1]. The disease is characterized by high case‐fatality rates ranging from 25% to 90%, depending on the Ebola virus species involved [2]. EVD is transmitted through direct contact with the blood, secretions, organs, or other bodily fluids of infected persons, and through contact with contaminated surfaces and materials [3].

EVD remains a significant public health threat in sub‐Saharan Africa since its initial identification in 1976, following two epidemics that occurred approximately 800 km apart in Nzara (southern Sudan) and Yambuku (northern Zaire) [1, 4]. The largest EVD outbreak occurred in West Africa from 2013 to 2016, with 28,652 confirmed cases and 11,325 deaths reported (case‐fatality rate: 39.5%), whereas the second‐largest outbreak occurred in the Democratic Republic of the Congo (DRC), primarily in North Kivu and Ituri provinces [5]. Uganda has experienced multiple outbreaks and faces increased vulnerability due to refugee influxes from the DRC and Sudan, with the most recent being its eighth outbreak in 2025 [4]. The disease’s high case‐fatality rate, potential for rapid transmission, and the necessity for stringent infection prevention and control (IPC) measures underscore the critical importance of a well‐prepared and responsive health system. Health facilities serve as frontline structures for the early detection, management, and containment of EVD outbreaks [6]. However, systemic challenges such as inadequate infrastructure, limited IPC capacity, insufficient staff training, and shortages of essential supplies have historically impeded timely and effective responses [7].

Jinja was identified as one of the country’s EVD hotspots, having experienced two EVD outbreaks: the first in November 2022 and the second in February 2025. During the most recent outbreak, three confirmed EVD cases were reported, all epidemiologically linked to the index case: a 32‐year‐old male nurse from Mulago National Referral Hospital (unpublished Jinja EVD situation report 2025).

Therefore, assessing the readiness of healthcare facilities in Jinja was essential for identifying existing preparedness gaps and informing targeted interventions. Such assessments are crucial for strengthening emergency response capacity, enhancing facility‐level resilience, safeguarding healthcare workers, and facilitating the rapid containment of future EVD outbreaks.

## 2. Materials and Methods

### 2.1. Study Design

A cross‐sectional study integrating both qualitative and quantitative approaches was conducted purposively in 36 high‐volume healthcare facilities in Jinja District in April 2025. These facilities included one district health office, three hospitals, one health centre IV, seven health centers III, and 24 health centers II, as well as medical centers.

The World Health Organization (WHO) EVD readiness checklist was used to evaluate facility preparedness across multiple domains, including IPC, surveillance and early detection, triage and isolation, case management, laboratory services, risk communication and community engagement, logistics and supply chain, and staff training and simulation exercises [8].

### 2.2. Study Area

**FIGURE 1:**
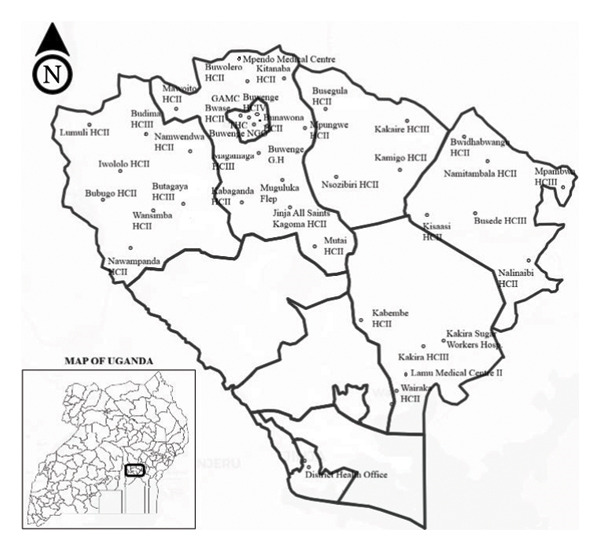
Map of Jinja district, Eastern Uganda, showing the distribution of high‐volume health facilities assessed for Ebola virus disease readiness and response (*n* = 36). GAMC: Guardian Angel Medical Clinic. THC: Teso Health Facility. EVD: Ebola virus disease. HC II: health centre level two. HC III: health centre level three. HCIV: health centre level four. NGO: nongovernmental organization.

Jinja District is situated in eastern Uganda, along the northern shores of Lake Victoria, near the source of the River Nile, approximately 80 km east of the national capital, Kampala. It serves as both an industrial and tourism hub. Kamuli District borders Jinja to the north, Buikwe District to the south, Kayunga District to the west, Luuka District to the northeast, and Mayuge District to the east, with Jinja City serving as the main industrial hub. Its proximity to Lake Victoria and the River Nile, combined with industrial activity and tourism, has implications for both economic development and public health, particularly by increasing the risk of disease transmission due to heightened population movement (Figure 1).

An assessment of 36 high‐volume healthcare facilities in Jinja District was conducted using the WHO EVD readiness checklist, evaluating seven key domains: coordination, surveillance, case management & IPC, community engagement, supplies & logistics, laboratory systems, and overall readiness/response (Figure 1).

### 2.3. Data Collection and Analysis

Data were collected through facility walkthroughs, key informant interviews, and discussions with facility staff. Document reviews were also conducted, including the examination of standard operating procedures (SOPs), case definitions, training records, and reporting systems.

Quantitative data were collected using a structured electronic questionnaire administered via the Kobo Collect platform. Data were exported to Microsoft Excel and analyzed using R version 4.5.0, with facility attributes scored across predefined domains, including coordination, surveillance, case management, and IPC, risk communication and community engagement, logistics and supplies, and laboratory systems. Quantitative data were analyzed by scoring readiness indicators and categorizing performance as not started, in progress, or complete to facilitate prioritization of preparedness levels. Readiness indicators were scored and categorized as not started, in progress, or complete to facilitate prioritization of preparedness levels. Qualitative data were collected through key informant interviews with 37 purposively selected participants, including two District Health Team members and 35 health facility managers comprising diploma‐level clinical officers, diploma nurses, and one medical officer. Interviews explored EVD preparedness across predefined thematic domains. Audio‐recorded interviews were transcribed verbatim and analyzed using a thematic approach, focusing on readiness categorization, performance across domains, and identification of key gaps, strengths, and enabling factors.

### 2.4. Ethical Considerations

Ethical approval was obtained from the relevant institutional review board of Jinja Regional Referral Hospital, and the study adhered to WHO research guidelines. Permissions were secured from the national EVD coordination pillar, and informed consent was obtained from all participants, with confidentiality maintained throughout.

## 3. Results

### 3.1. Pillar Scores

**FIGURE 2:**
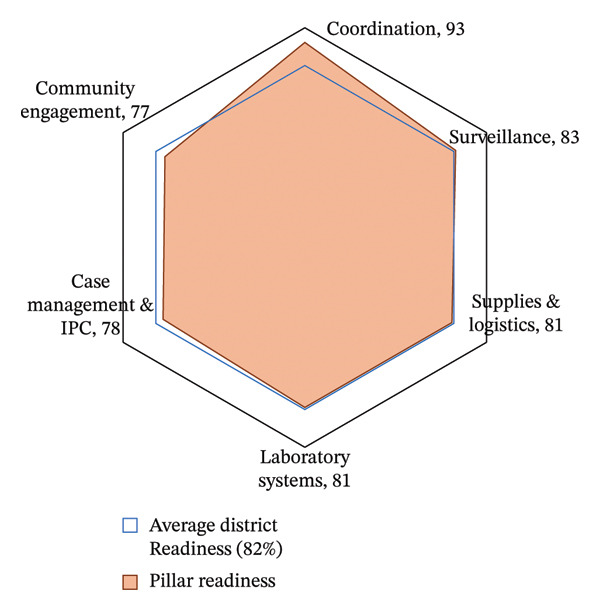
Average Ebola virus disease preparedness scores across key response domains among 36 high‐volume health facilities in Jinja district, Uganda. IPC: infection prevention control.

A radar chart summarized the average scores of all assessed healthcare facilities across six EVD readiness pillars, illustrating the relative performance of each domain against the district readiness benchmark of 82% (Figure 2).

Coordination scored highest at 93%, indicating the presence of active task forces, clearly defined leadership roles, and well‐established coordination protocols. Surveillance achieved a score of 83%, slightly exceeding the district threshold, indicating a relatively strong capacity for case detection and reporting. Supplies & Logistics and Laboratory Systems each scored 81%, indicating moderate capacity in personal protective equipment (PPE) availability and laboratory systems functionality. Case Management & IPC scored 78%, highlighting gaps in standard IPC implementation and limitations in infrastructure for patient isolation and treatment. Community engagement recorded the lowest score at 77%, underscoring ongoing challenges in community linkages and risk communication strategies (Figure 2).

### 3.2. Overall Readiness and Categorization

A total of 36 healthcare facilities were assessed for their readiness to respond to EVD. Facilities were categorized into three levels based on their overall scores across key thematic areas. The EVD readiness response indicator scores were classified as high readiness (> 80%), medium readiness (60%–79%), and low readiness (< 59%) (Table 1).

**TABLE 1:** Summary of Ebola virus disease (EVD) health facility readiness and response categorization scores.

Readiness level	Number of facilities (*n*)	Percentage (%)
High readiness (> 80%)	20	55.6
Medium readiness (60%–79%)	7	19.4
Low readiness (< 59%)	9	25.0

*Note: n*: counts.

Of the 36 healthcare facilities evaluated, 20 (55.6%) demonstrated high readiness, 7 (19.4%) exhibited medium readiness, and 9 (25%) showed low readiness (Table 1 and Figures 3(a), 3(b), and 3(c)).

FIGURE 3Summary of Ebola virus disease (EVD) readiness response scores among 36 high‐volume healthcare facilities in Jinja. (a) Healthcare facilities with high Ebola virus disease readiness and response scores. (b) Healthcare facilities with medium Ebola virus disease readiness and response scores. (c) Healthcare facilities with low Ebola virus disease readiness and response scores. PNFP: private not‐for‐profit.

**Figure:**
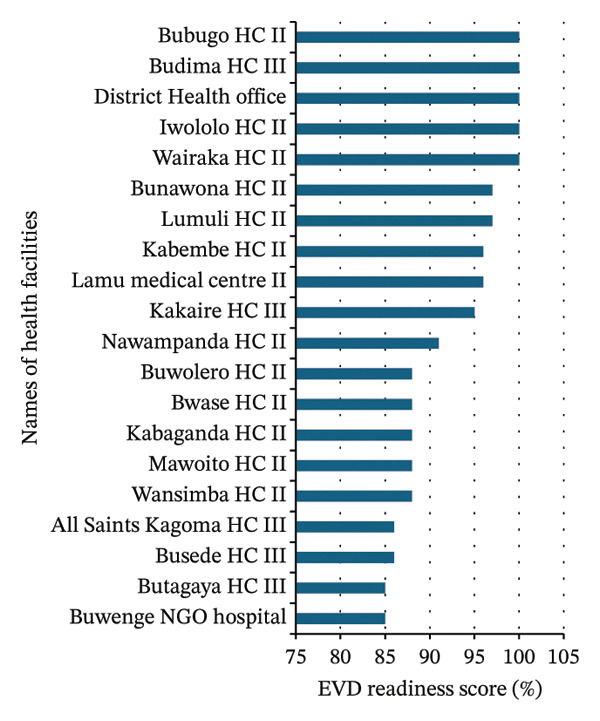
(a)

**Figure:**
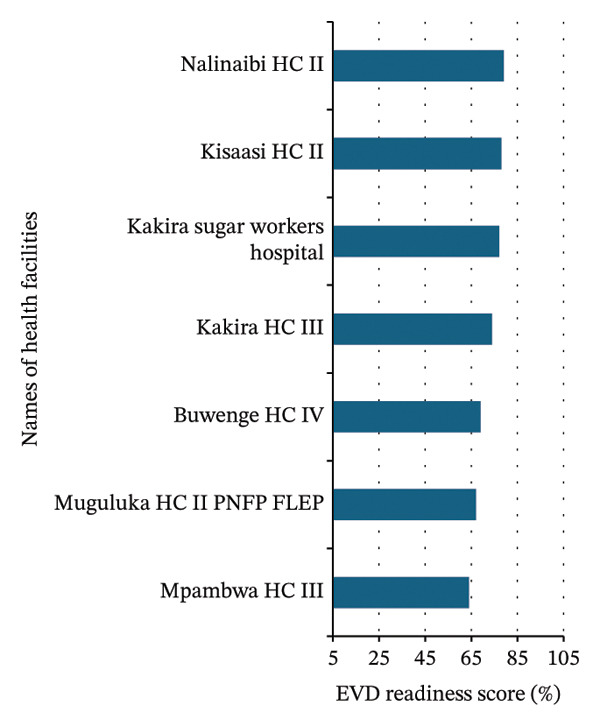
(b)

**Figure:**
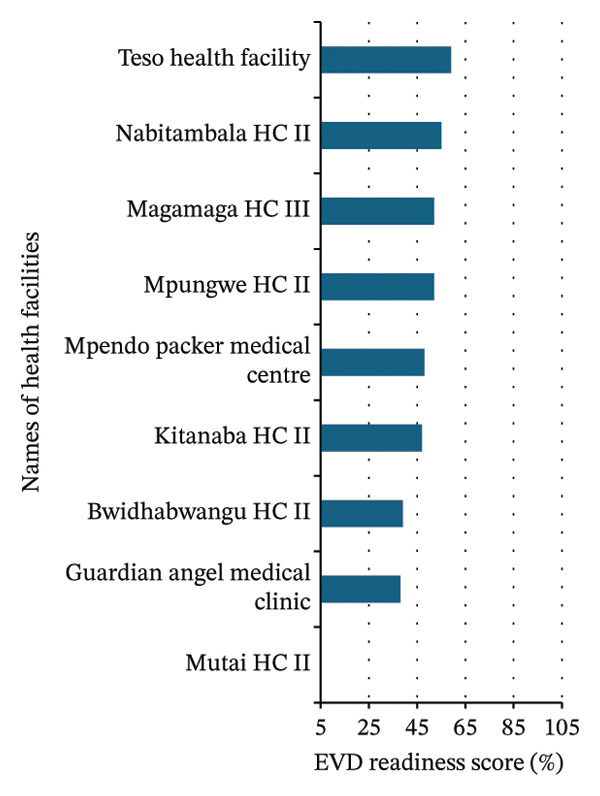
(c)

More than half of the healthcare facilities demonstrated high EVD readiness, indicating a generally strong level of preparedness. However, 44.4% of the facilities showed moderate to critical gaps, highlighting the need for targeted improvements in specific areas (Figures 3(a), 3(b), and 3(c)).

### 3.3. Healthcare Facilities Readiness and Response Categorization

The best‐performing facilities—including the District Health Office, Wairaka HCII, Iwololo HCII, Budima HCIII, and Bubugo HCII—consistently scored high across all domains (Figure 3(a) and Table 2). In contrast, the medium‐performing facilities—including Nalinaibi HCII, Kisaasi HCII, Kakira Sugar Workers Hospital, Kakira HCIII, Buwenge HCIV, Muguluka FLEP HCII, and Mpambwa HCIII—demonstrated moderate scores, often due to domain‐specific weaknesses (Figure 3(b) and Table 2). However, the low‐performing facilities—including Nabitambala HCII, Teso Healthcare, Mpungwe HCII, Magamaga HCIII, Mpendo Parkers Medical Centre, Kitanamba HCII, Bwidhabwangu HCII, Guardian Angel Medical Clinic, and Mutai HCII—exhibited persistent gaps in IPC, logistics, and laboratory readiness (Figure 3(c) and Table 2).

**TABLE 2:** Summary of Ebola virus disease (EVD) health facility readiness and response performance across thematic domains.

No.	Facility	Coordination	Surveillance	Case management & IPC	Community engagement	Supplies & logistics	Laboratory system	Overall readiness/response
1	All Saints Kagoma HCIII	High	High	High	High	High	Medium	High
2	Bubugo HCII	High	High	High	High	High	High	High
3	Budima HCIII	High	High	High	High	High	High	High
4	Bunawona HCII	High	High	High	High	High	High	High
5	Busede HCIII	High	High	High	Medium	High	High	High
6	Butagaya HCIII	High	High	High	Medium	High	High	High
7	Buwenge HCIV	High	Medium	High	Medium	Medium	High	Medium
8	Buwenge NGO Hospital	High	High	High	High	High	Medium	High
9	Buwolero HCII	High	High	High	High	High	High	High
10	Bwase HCII	High	High	High	High	High	High	High
11	Bwidhabwangu HCII	High	Low	Medium	Low	Medium	Low	Low
12	District Health Office	High	High	High	High	High	High	High
13	Guardian Angel Clinic	High	Low	Medium	Low	Medium	Low	Low
14	Iwololo HCII PNFP	High	High	High	High	High	High	High
15	Kabaganda HCII	High	High	High	High	High	High	High
16	Kabembe HCII	High	High	High	High	High	High	High
17	Kakaire HCIII	High	Medium	High	High	Medium	High	Medium
18	Buwenge General Hosp.	High	High	High	High	High	High	High
19	Kakira Workers Hospital	High	Medium	High	High	Medium	Medium	Medium
20	Kisaasi HCII	High	High	High	High	High	Low	Medium
21	Kitanaba HCII	High	Low	Medium	Medium	Medium	Low	Low
22	Lamu Medical Center II	High	High	High	High	High	High	High
23	Lumuli HCII	High	High	High	High	High	High	High
24	Magamaga HCIII	High	Low	Medium	High	Medium	Low	Low
25	Mawoito HCII	High	High	High	High	High	High	High
26	Mpambwa HCIII	High	Low	Medium	Medium	Medium	High	Medium
27	Mpendo Paker Medical Clinic	High	Low	Medium	Medium	Medium	Low	Low
28	Mpungwe HCII	High	Low	Medium	High	Medium	Low	Low
29	Muguluka HCII PNFP	High	Low	High	High	High	Low	Medium
30	Mutai HCII	Low	Low	Low	Low	Low	Low	Low
31	Nabitambala HCII	High	Medium	Medium	High	Medium	Low	Low
32	Nalinaibi HCII	High	High	High	High	High	Low	Medium
33	Nawampanda HCII	High	High	High	Medium	High	High	High
34	Teso Healthcare Facility	High	Medium	High	Medium	Medium	Low	Low
35	Wairaka HCII	High	High	High	High	High	High	High
36	Wansimba HCII	High	High	High	High	High	High	High

*Note:* HC II: health center level two. HC III: health center level three. HCIV: health center level four. Hosp: hospital. High: high EVD readiness score. Medium: medium EVD readiness score with limited gaps. Low: low EVD readiness score with significant gaps.

Abbreviations: IPC, infection prevention and control; NGO, nongovernmental organization; PNFP, private not‐for‐profit.

### 3.4. Thematic Domain Performance

#### 3.4.1. Coordination

Coordination capacity was strong across the district, with 35 out of 36 facilities (97.2%) achieving high scores (Table 2). This reflects the presence of active District Task Forces (DTF), clearly defined roles, and functional leadership and supervision structures at the facility level. However, lower‐level facilities demonstrated weak and irregular coordination mechanisms (Table 3).

**TABLE 3:** Results of the thematic analysis of key informant interviews (KIIs).

Themes	Emergent themes from KIIs	Key gaps identified	Preparedness implications
Coordination	• Presence of health facility surveillance focal persons • Irregular coordination mechanisms at facilities	Weak functionality of facility coordinators; unclear reporting lines; limited supervision; incomplete plans	Delayed decision‐making and fragmented response during alerts

Surveillance	• Operational surveillance systems and guidelines • Weak feedback mechanisms	Underutilization of VHT; lack of CHEW; poor case recording and reporting; limited training	Reduced early detection and delayed outbreak notification

Case management & IPC	• Variable IPC adherence • Limited isolation capacity at facilities	Lack of isolation space; PPE stockouts; limited IPC training; poor waste management	Increased risk of healthcare‐associated transmission undermines preparedness

Risk communication & community engagement	• Reliance on one‐way information dissemination • Limited community linkage	Limited community engagement and feedback mechanism; limited IEC materials; lack of CHEW	Delayed community compliance with prevention and control measures

Logistics & supplies	• Intermittent availability of essential IPC supplies	PPE stock‐outs, outdated stock records, and broken referral systems	Interruptions in safe care and response continuity

Laboratory systems	• Limited laboratory functionality at HCII and HCIII level	Few trained personnel; limited sample collection capacity; delayed transport and timely reporting	Delayed case confirmation and weakened surveillance sensitivity

*Note:* IEC: information, education, and communication materials. HC II: health center level two. HC III: health center level three.

Abbreviations: CHEW, community health extension workers; IPC, infection prevention and control; PPE, personal protective equipment; VHT, village health teams.

#### 3.4.2. Surveillance

Surveillance performance was high in 22 facilities (61.1%), indicating effective case detection and reporting systems (Table 2). However, 14 facilities (38.9%) demonstrated medium or low performance, largely due to deficiencies in timely case identification, incomplete reporting, and limited availability of trained personnel (Table 3).

#### 3.4.3. Case Management & IPC

IPC performance was generally high, with 27 facilities (75.0%) scoring in the high category (Table 2). However, 9 facilities (25.0%) scored medium or low, primarily due to inconsistent implementation of IPC (including weak or absent IPC practices and inadequate PPE), limited case management infrastructure (i.e., lack of isolation facilities), and gaps in waste management practices (Table 3).

#### 3.4.4. Community Engagement

This domain showed the greatest variation in performance. While many facilities scored high, 8 facilities (22.2%) scored medium, and 3 facilities (8.3%) scored low (Table 2). Key challenges included reliance on one‐way information dissemination and limited community linkage (Table 3).

#### 3.4.5. Supplies & Logistics

Most facilities (23, 63.9%) scored high in this domain (Table 2). However, 13 facilities (36.1%) recorded medium or low performance, highlighting challenges related to intermittent availability of essential IPC supplies (Table 3).

#### 3.4.6. Laboratory Systems

Fifteen facilities (41.7%), particularly health centers II and III, scored medium or low in this domain (Table 2). These scores reflect key limitations in laboratory functionality, especially at health centre II and health center III levels, including a few trained personnel, limited sample collection capacity, delayed transportation, and untimely laboratory reporting (Table 3).

## 4. Discussion

This investigation into the readiness of healthcare facilities in Jinja District for EVD response revealed that while over half of the facilities (55.6%) were highly prepared, significant readiness gaps remain in community engagement, IPC, laboratory systems, and supply logistics. These findings mirror earlier evidence from Ebola outbreaks in West Africa and Uganda, which emphasized that weak health system infrastructure and unprepared peripheral facilities severely compromise outbreak response capacity [9–11].

The uniformly high performance in coordination (100% of facilities) reflects an effective district‐level response architecture, likely shaped by prior outbreak experiences and national EVD contingency planning [12, 13]. This aligns with literature that identifies multisectoral coordination, clear leadership, and effective supervision as pillars for rapid public health emergency responses [14, 15]. Similarly, the strong showing in surveillance (86.1% high performers) suggests improved health information systems, trained surveillance teams, and timely reporting mechanisms, consistent with successful case detection systems seen in Liberia and Nigeria during past outbreaks [16, 17]. However, surveillance readiness at peripheral facilities was compromised by limited training, resulting in suboptimal case recording.

Despite national investments in IPC training and infrastructure following past outbreaks, 25.0% of facilities reported medium and low performance, with significant implementation gaps in this study [18]. Inconsistent IPC practices observed across health facilities were driven by a combination of workforce, supply, and system‐level constraints. Limited IPC training contributed to variable adherence to standard precautions, particularly in lower‐level facilities. These challenges were also exacerbated by the intermittent availability of essential IPC supplies, including PPE and hand hygiene materials, leading to selective compliance rather than routine practice. Inadequate infrastructure, such as unreliable water supply, poor waste management systems exacerbated by the withdrawal of funding that supported out‐of‐site disposal, and lack of isolation space, further constrained effective IPC implementation. Additionally, weak governance mechanisms, including the absence of functional IPC committees and irregular supportive supervision, reduced accountability and corrective action. Collectively, these gaps heighten the risk of healthcare‐associated infections and undermine preparedness. These challenges were more pronounced in lower‐level health facilities, where high workload and limited managerial oversight further compromised adherence to standard IPC protocols. Previous studies in the DRC confirm that IPC breaches and poor health worker protection have contributed to nosocomial Ebola transmission in resource‐limited settings [19, 20]. The lack of adaptation of IPC protocols to the local context could affect implementation and may have contributed to the observed suboptimal IPC performance.

The assessment revealed that community engagement was the most critical gap, with several health facilities demonstrating weak involvement of village health teams (VHTs), inadequate feedback mechanisms, and limited dissemination of information, education, and communication (IEC) materials. Integrating CHEWs alongside VHTs could strengthen community linkages and enhance preparedness for future outbreaks. Similarly, literature from the 2014–2016 West Africa Ebola outbreak highlighted that mistrust, poor risk communication, and lack of culturally appropriate outreach were key drivers of resistance to public health interventions [21, 22]. These findings reaffirm that without effective community partnerships, even technically strong responses risk failure.

Although most facilities maintained adequate stocks, eight facilities (22%) reported gaps in PPE availability, outdated stock records, and broken referral systems. Stock‐outs and delayed supply chains have repeatedly been identified as persistent challenges in Uganda’s health sector, particularly in emergency contexts [23].

Laboratory readiness was also suboptimal, with nearly half of the assessed facilities (41.7%), mainly health centers II and III, demonstrated medium to low performance in the laboratory systems. Compared with higher‐level facilities, these lower‐level health centers exhibited more pronounced limitations in sample collection, specimen transportation, and delays in laboratory result reporting, constraining timely case confirmation and weakened surveillance sensitivity highlighting persistent capacity gaps at peripheral levels of care. The observed gaps in laboratory capacity for sample collection are multifactorial, stemming from shortages of trained personnel, limited technical knowledge and skills, and inadequate material and infrastructure support. Addressing these gaps requires an integrated approach targeting human resource development, continuous capacity building, and the provision of essential materials and logistics. In other studies, laboratory readiness was also suboptimal, especially among HCIIs and HCIIIs, which lacked capacity for sample collection and reporting, an issue that impedes early case confirmation and outbreak containment [24]. These deficiencies may delay outbreak detection and response, especially in decentralized health systems where lower‐level facilities serve as the first point of contact for suspected cases.

The disparities in readiness between higher‐level health centers and peripheral HC IIs/IIIs underscore the need for tailored interventions based on facility capacity. Regular simulation exercises, improved laboratory networks, and stock buffer systems are essential to reinforce frontline preparedness. This assessment also supports WHO’s recommendation that public health emergency preparedness must be decentralized, inclusive, and continuously assessed [25].

The identification of low and medium readiness in over 44% of facilities highlights the need for focused investment in specific areas such as IPC, community engagement, logistics, and laboratory capacity. Policies should prioritize resource allocation based on risk and performance gaps to optimize emergency preparedness. This is consistent with findings from a study on Uganda’s experience with health facility COVID‐19 readiness assessments, which underscores the critical need for focused investment in foundational components of health system preparedness. The study identifies IPC and logistics as key pillars that must be strengthened to enable a timely and effective response to public health emergencies [26].

The disparity in readiness scores highlights the need for standardized national or regional benchmarks for EVD preparedness. Health authorities should enforce minimum readiness standards and integrate them into routine supervision, licensing, as part of broader health systems resilience monitoring, and facility performance reviews. A growing body of evidence supports the importance of standardized and enforced benchmarks to reduce disparities in facility preparedness. According to WHO guidelines, health authorities are encouraged to establish such minimum standards at national or subnational levels. These benchmarks can be embedded within supervision checklists, licensing processes, and routine performance assessments. Enforcement mechanisms—such as linking compliance to accreditation or funding—can further incentivize sustained facility readiness [27].

The strong performance in coordination across facilities highlights the value of active DTFs. Public health authorities should institutionalize these coordination mechanisms by providing legal and operational backing to ensure their continuity, even outside of outbreak periods. Retrospective analyses using preparedness frameworks have confirmed that DTF activation was pivotal in maintaining consistent preparedness planning and execution across different risk categories. Institutionalizing these structures—through legal mandates and formal operational procedures—would embed them into routine health system functions, strengthening district‐level capacity to respond swiftly and cohesively to emerging health threats [12].

The weaker performance in community engagement highlights the need for policies that strengthen risk communication and social mobilization. Investing in VHTs, local leaders, adoption of the community health extension workers (CHEWs) approach, and culturally tailored communication can foster community trust and support early detection and response efforts. A 2020 population‐based survey conducted in six Ugandan districts found that, although exposure to EVD messages was significantly higher in high‐risk areas, overall community perceptions and preventive practices remained suboptimal, particularly in districts with weaker engagement strategies [28]. This suggests that simply disseminating information is not enough; trust, cultural relevance, and community agency are crucial components.

Gaps in logistics and supply chain management suggest a need for reforms in procurement, stock monitoring, and emergency supply prepositioning. Policies should mandate buffer stocks of PPE and critical supplies in high‐risk districts, with digital systems to track usage and replenishment. Assessments in Kasese and Rubirizi districts also revealed that over half of health facilities lacked the logistics infrastructure—and often PPE—that would be immediately needed in an Ebola outbreak, indicating a vulnerability that must be addressed through proactive policy action [7].

The findings suggest that training and simulation exercises should be routine rather than reactive. Public health policy should support continuous professional development, regular emergency drills, and integrated mentorship programs to sustain a skilled and responsive healthcare workforce. Evidence from studies indicates that simulation‐based approaches—such as low‐dose, high‐frequency training—have been effective in Ugandan contexts, including maternal and emergency care [29]. The brief, frequent drills have been shown to reinforce clinical competencies and build provider confidence.

### 4.1. Study Strengths

The use of a standardized WHO checklist, combined with multiple data collection methods, allowed for a comprehensive and reliable assessment of EVD readiness. The focus on high‐volume facilities ensured relevance to outbreak response, and the findings generated actionable recommendations to guide preparedness interventions.

### 4.2. Study Limitations

This assessment had limitations. The cross‐sectional design captured readiness at a single time point, potentially overlooking ongoing changes. Reliance on self‐reported data and observations may have introduced recall, social desirability, and observer biases. While the WHO EVD checklist offered a standardized approach, it may not have fully accounted for the local context. The focus on 36 high‐volume facilities in Jinja District limits generalizability to other settings. Additionally, time and resource constraints may have restricted the depth and validation of the data collected.

## 5. Conclusion

In conclusion, the assessment of EVD readiness across six critical pillars in Jinja District revealed a generally strong preparedness landscape, particularly in coordination, where nearly all facilities demonstrated high performance. Surveillance and IPC also showed encouraging results, though notable gaps remain in a subset of facilities, driven by deficiencies in reporting systems, inconsistent IPC practices, and limited infrastructure. Community engagement emerged as the most variable domain, with several facilities struggling with VHT integration and effective risk communication. Similarly, significant weaknesses were observed in laboratory systems and supply chain management, particularly among lower‐level facilities, indicating systemic challenges in specimen handling, reporting, and logistics. These findings underscore the need for targeted support to strengthen underperforming areas, especially community engagement, laboratory capacity, and supply chain systems, to ensure a uniformly high level of readiness across all healthcare facilities in the district. Enhancing these areas will be critical for enabling a timely, coordinated, and effective response to future EVD outbreaks.

To enhance health facility readiness, targeted support should be prioritized for underperforming facilities through mentorship, regular supervision, and structured improvement plans. Functional Sub‐County Task Forces and facility‐level emergency teams should be reactivated or established in low‐ and medium‐performing areas. IPC practices must be standardized, with consistent availability of PPE and designated isolation or holding areas. Community engagement efforts should be strengthened through VHT training, dissemination of IEC materials, and implementation of responsive feedback mechanisms. Strengthening supply chain systems—including buffer stock management, timely distribution, and accurate record‐keeping—is essential. Ongoing assessments and supportive supervision are also necessary to monitor progress and sustain high levels of readiness across all facilities.

## Funding

No funding was received for this study.

## Conflicts of Interest

The authors declare no conflicts of interest.

## Data Availability

The data that support the findings of this study are available upon request from the corresponding author. The data are not publicly available due to privacy or ethical restrictions.
